# Ocular safety of 222‐nm far‐ultraviolet‐c full‐room germicidal irradiation: A 36‐month clinical observation

**DOI:** 10.1111/php.14052

**Published:** 2024-12-10

**Authors:** Kazunobu Sugihara, Sachiko Kaidzu, Masahiro Sasaki, Sho Ichioka, Ichiya Sano, Katsunori Hara, Masaki Tanito

**Affiliations:** ^1^ Department of Ophthalmology Shimane University Faculty of Medicine Izumo Japan; ^2^ Ushio Inc. Tokyo Japan

**Keywords:** 222 nm excimer lamp, full‐room ultraviolet germicidal irradiation (UVGI), health hazard, clinical trial, ocular safety

## Abstract

The ocular safety of 222‐nm far‐ultraviolet‐C (UV‐C) irradiation, widely recognized for its germicidal properties, was evaluated in a clinical setting to assess its long‐term health effects on the human eye. This prospective observational study involved a 36‐month follow‐up of physicians working in an ophthalmic examination room equipped with 222‐nm UV‐C lamps. Initially, a 12‐month observation showed no signs of acute or chronic ocular damage. To further substantiate these findings, the study period was extended to 36 months, during which four participants underwent regular ocular examinations, including assessments of visual acuity, refractive error, and corneal endothelial cell density. The irradiation dose was meticulously controlled to remain within the previous threshold limit of 22 mJ/cm^2^ over an 8‐h period, as advised by the ACGIH prior to 2022. Results indicated no significant changes in these parameters, suggesting no clinically significant ocular hazards associated with prolonged exposure to 222‐nm UV‐C irradiation under real‐world conditions. Additionally, no delayed side effects, such as pterygium, keratopathies, or cataracts, were observed. Our study supports the safe use of 222‐nm UV‐C for microbial disinfection in occupied environments and provides a robust foundation for updated safety guidelines.

## Graphical abstract

A 36‐month observational study evaluated the ocular safety of 222‐nm UV‐C irradiation in physicians exposed in an ophthalmic room. No acute or chronic ocular damage was detected, and key ocular parameters remained unchanged. The findings support the safe use of 222‐nm UV‐C for disinfection, aligning with safety threshold guidelines.

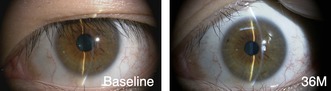

**Abbreviations**
ACGIH: the American Conference of Governmental Industrial Hygienists
BCVA: best‐corrected visual acuity
CECD: corneal endothelial cell density
IRB: institutional review board
KrBr: krypton‐bromide
KrCl: krypton‐chloride
LogMAR: logarithm of minimum angle of resolution
SERE: spherical equivalent refractive error
TLV: threshold limit value
UV‐B: ultraviolet‐B
UV‐C: ultraviolet‐C
UBGI: full‐room ultraviolet germicidal irradiation

## INTRODUCTION

Ultraviolet (UV) radiation covers the wavelength range from 100 nm to 400 nm and is divided into photobiological subcategories: UV‐C (100–280 nm), UV‐B (280–315 nm), and UV‐A (315–400 nm). Among these, UV‐C is particularly effective for disinfection, showing strong antiviral and antibacterial properties.^1^, ^2^, ^3^ However, conventional 254‐nm UV‐C germicidal devices are typically restricted to unoccupied areas, such as upper room air, in human‐occupied environments due to the risk of skin irritation (erythema) and eye injury (photokeratitis) from direct exposure at this wavelength.^2^, ^3^

Recently, shorter‐wavelength ultraviolet germicidal irradiation (UVGI) sources, known as “far UV‐C,” such as krypton‐bromide (KrBr) and krypton‐chloride (KrCl) excimer lamps, which emit at 207 nm and 222 nm respectively, have attracted considerable interest. These sources enable whole‐room irradiation that is safe for human exposure while effectively deactivating microorganisms.^4^, ^5^, ^6^, ^7^ Studies on animals have shown that UV penetration depth in the corneal epithelium of rats varies significantly with wavelength. Far‐UV‐C wavelengths, like 207 nm and 222 nm, penetrate only the outermost cell layers of the corneal epithelium, which are naturally shed within about 24–48 h due to normal cell turnover.^8^ This limited penetration and rapid cell renewal may account for the reduced risks associated with far‐UV‐C wavelengths.^9^, ^10^, ^11^

The American Conference of Governmental Industrial Hygienists (ACGIH) initially established the threshold limit value (TLV) for 222 nm UV‐C exposure at 22 mJ/cm^2^/day,^2^ but this limit was later updated to 160 mJ/cm^2^/day.^7^, ^12^, ^13^ Research from half a century ago found that photokeratitis thresholds for wavelengths between 215 and 225 nm were 46 mJ/cm^2^ in rabbits, 21 mJ/cm^2^ in primates, and 10 mJ/cm^2^ in humans.^14^ More recent studies using rat models with high‐output KrBr and KrCl excimer lamps indicated that the minimum photokeratitis threshold doses for 207 nm and 222 nm were 15,000 and 5000 mJ/cm^2^, respectively.^10^ As a result, the ACGIH's TLV remains considerably below the photokeratitis thresholds observed in rat studies, yet it exceeds the dose levels identified in earlier human‐based research.

To evaluate the ocular safety of UV‐C lamps for human exposure, we recently conducted a human study involving exposure to 222 nm far UV‐C light.^15^ The study indicated that exposure up to 75 mJ/cm^2^ of 222‐nm far UV‐C does not lead to “clinically significant photokeratitis,” although it may cause mild, temporary discomfort immediately following exposure.^15^ These results support the use of 222‐nm UV‐C in germicidal applications within occupied spaces and provide a foundation for updated safety guidelines. A key application of full‐room UVGI is in clinical settings, such as during ophthalmic exams where the physician is positioned directly in front of the patient. In July 2020, we installed 222‐nm lamp units in an examination room of our ophthalmology outpatient clinic and began a prospective, 1‐year observational study on eye safety for the staff working in that environment. Over the course of a year, the study reported no acute or chronic health issues in participants, while effectively reducing bacterial and phage presence in the room.^16^ Currently, there is limited literature on long‐term and real‐world assessments of human eye safety for far UV‐C UVGI. This report provides safety assessment findings observed over a 36‐month period.

## MATERIALS AND METHODS

### Study design

This research was designed as a prospective observational study involving human participants in the outpatient examination room of Shimane University Hospital's Department of Ophthalmology. The study complied with the principles outlined in the Declaration of Helsinki and adhered to Japan's Ethical Guidelines for Medical and Health Research Involving Human Subjects. The study protocol received initial review and approval from the Institutional Review Board at Shimane University Hospital (IRB No. 20200517‐2, approved on July 20, 2020), with participant follow‐up lasting 12 months. The original study observed six male ophthalmologists who were anticipated to spend over 4 h/week in the room, and the results were previously reported.^16^ Of these six participants, four continued working in the same room, prompting a new 36‐month observation under an updated protocol (IRB No. 20230608‐1, approved on July 18, 2023). All four participants provided written consent to continue. The average age of the participants was 41.8 ± 8.5 years, with three of the four wearing glasses for myopia correction; none used contact lenses. Simulations using computer‐aided design and experiments with a mannequin head indicated that corneal UV exposure remained largely similar with or without glasses, though glasses might shield the lower eyelid from UV exposure.^16^

### UV‐C lamps installation

**FIGURE 1:**
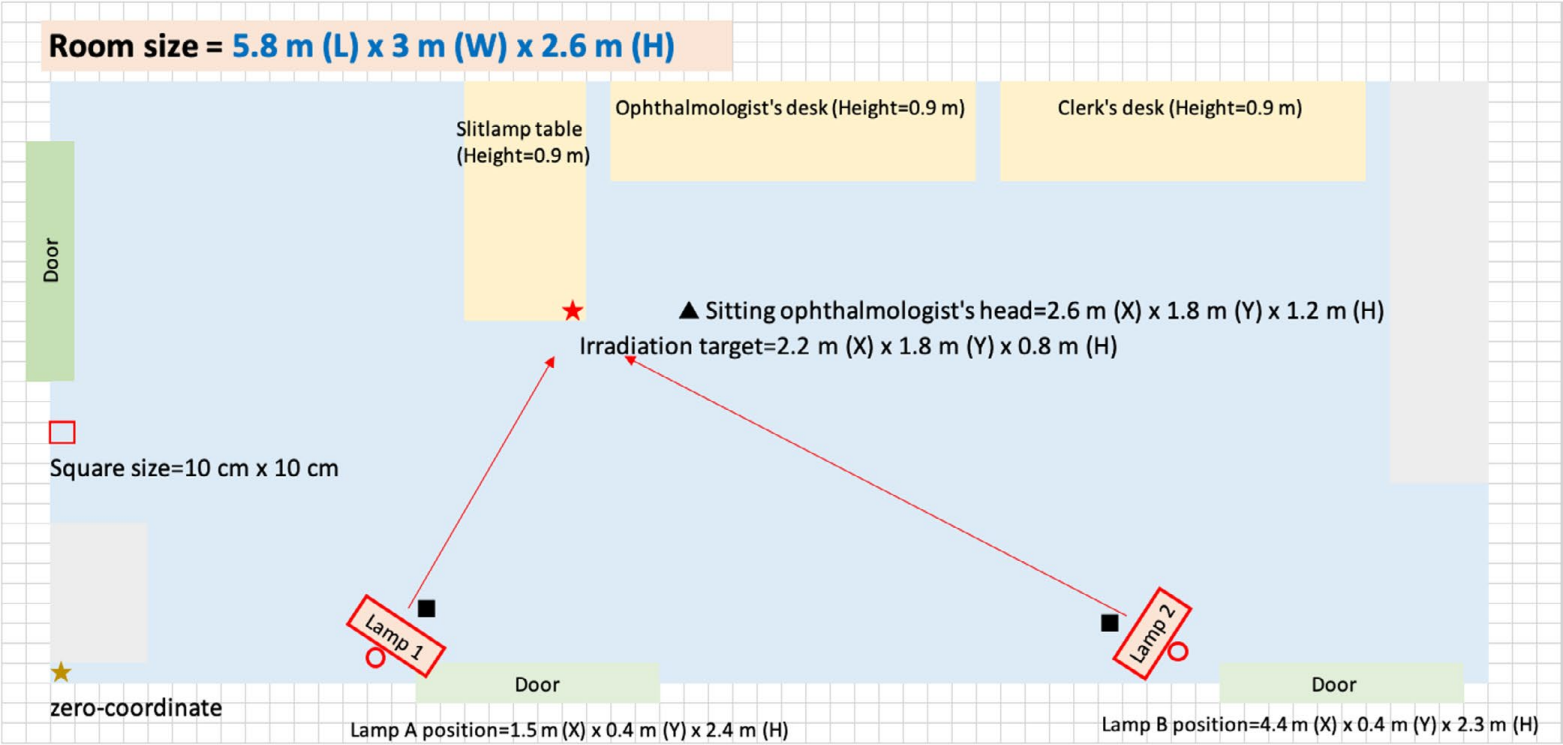
Schematic drawing of the Ophthalmology Department's examination room where the 222‐nm UV‐C lamps installed.

**FIGURE 2:**
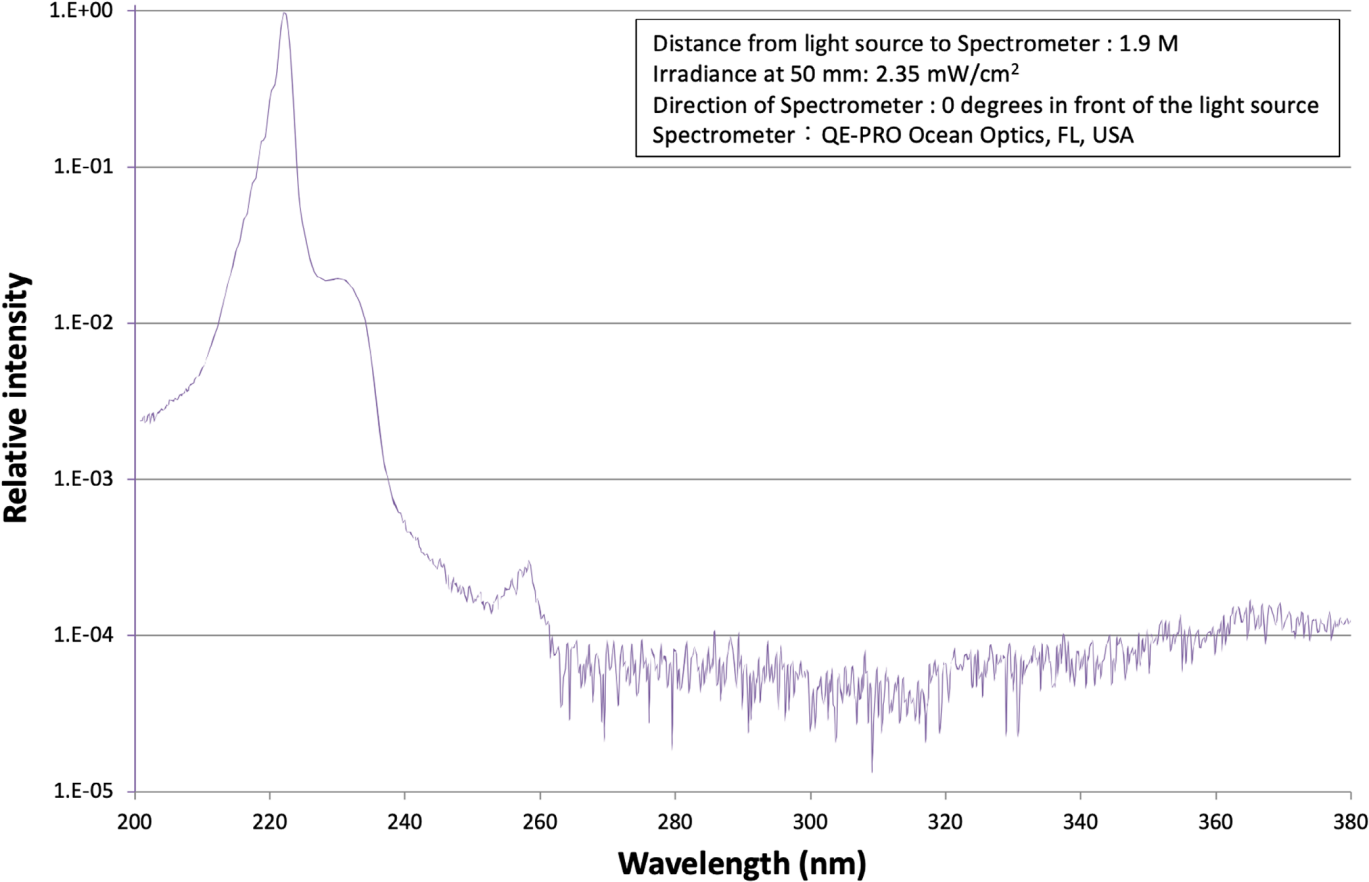
Spectral distribution of the 222‐nm far UV‐C lamps used in this study.

The specific setup of the lamps in the room has been previously outlined.^16^ In summary, two mercury‐free KrCl excimer lamp units (Care222 TRT‐104C11‐UI‐U3, USHIO Inc., Tokyo, Japan from July 2020 to December 2021 and Care222 iBT, USHIO Inc. from December 2021 onward) were installed in the outpatient clinic examination room for viral and bacterial inactivation (Figure 1, lamps 1 and 2). The spectral profile of the lamps, as measured by a spectrometer (QE‐PRO Ocean Optics, FL, USA), is presented in Figure 2. Each lamp emitted a peak wavelength of 222 nm and was fitted with a filter to block wavelengths above 230 nm, ensuring a complete cutoff beyond 240 nm. The units were mounted at heights of 240 cm (lamp 1) and 230 cm (lamp 2) above the floor, positioned to illuminate the slit lamp (Figure 1, red star) used in patient examinations. This configuration was intended to keep exposure levels within the previous threshold limit value (TLV) for 222 nm of 22 mJ/cm^2^, as recommended by the American Conference of Governmental Industrial Hygienists (ACGIH),^2^ assuming an individual of 170 cm in height could be exposed for 8 h directly beneath the lamps (Figure 1, black squares). Based on measurements (VUV‐S172/UIT‐250 USHIO INC.), when a participant (i.e., an ophthalmologist) was seated at the slit lamp facing a patient (Figure 1, black triangle), the maximum irradiance at the eye level of the participant was calculated to be approximately 0.002 mW/cm^2^.

### Examination schedule

**FIGURE 3:**
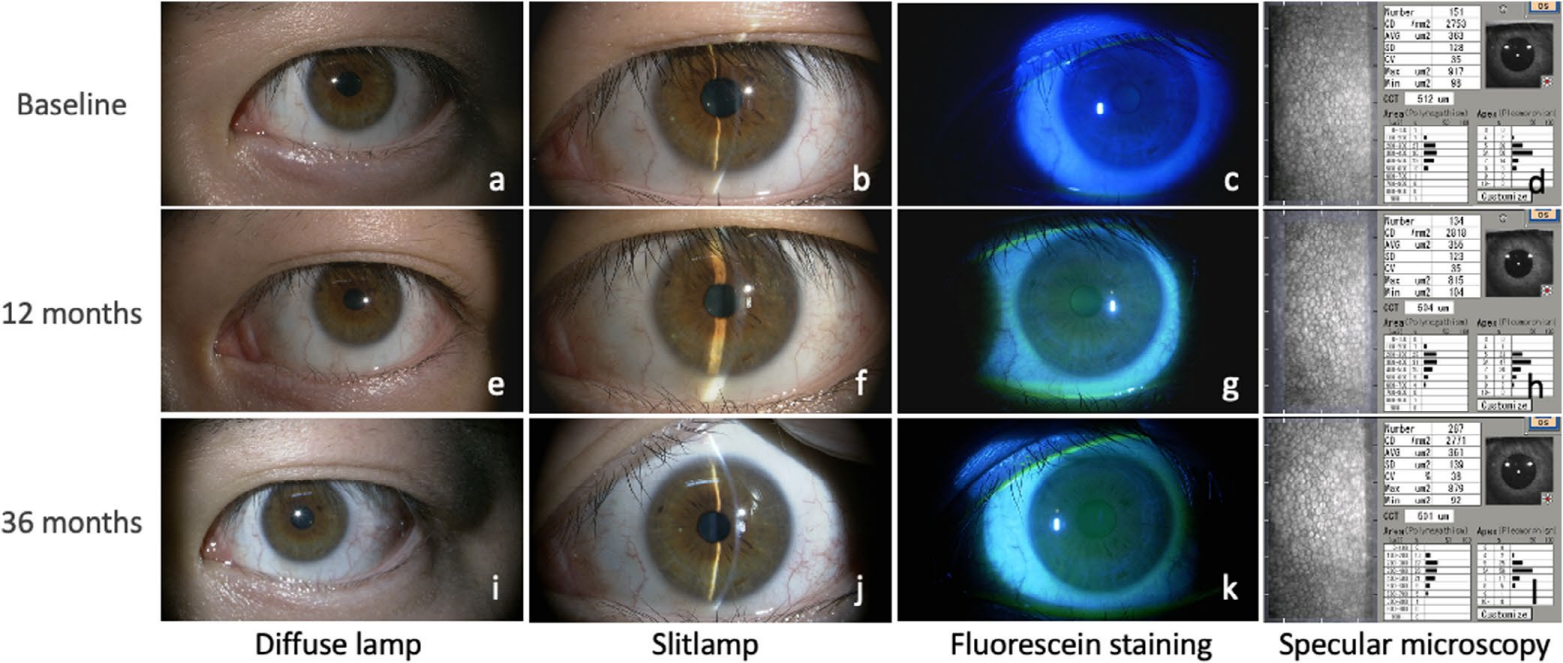
Representative images of slitlamp (A–K) and specular microscopic (D, H, L) observations at baseline (A–D), 12 months (E–H), and 36 months (I–L) (case 2, left eye).

To evaluate ocular safety, participants underwent examinations prior to starting work in the room (baseline), at the end of the first day (within 24 h of exposure), and at 1, 3, 6, 12, and 36 months after initiating work in the room. During each assessment, data on room exposure duration, bilateral best‐corrected visual acuity (BCVA), spherical equivalent refractive error (SERE), slit lamp findings, corneal endothelial cell density (CECD), subjective symptoms, and any potential adverse events were collected. SERE and CECD measurements were not conducted on the first day of examination. Participants reported their room exposure duration (per day on day one and weekly hours for subsequent periods). Visual acuity was evaluated using a decimal chart and converted to the logarithm of the minimum angle of resolution (LogMAR) for analysis. SERE was determined with an autorefractor‐keratometer (TonoRef III, Nidek, Gamagori, Japan). A slit lamp microscope (4ZL, Takagi, Nagano, Japan) was used to assess corneal erosion scores, conjunctival hyperemia scores, and to check for pterygium or cataract presence. It also documented any eyelid skin changes (erythema, pigmentation alterations) at 6.3× magnification (Figure 3A), whereas other findings were observed at 10× magnification (Figure 3B). Corneal erosion was visualized using sodium‐fluorescein staining under blue light (Figure 3C), and cataract evaluations involved both thin slit‐beam and diffuse lighting (Figure 3B). Corneal erosion scoring for each region was as follows: 0–3, with 0 indicating no punctate staining, 1 for less than one‐third coverage, 2 for one to two‐thirds, and 3 for more than two‐thirds of the cornea. Density scoring for superficial punctate keratopathy (SPK) ranged from 0 to 3: 0 for no staining, 1 for sparse density, 2 for moderate, and 3 for high density with overlapping lesions, as shown in reference images.^17^ Conjunctival hyperemia was graded per the Japanese Guidelines for Allergic Conjunctival Disease 2020, with score 0 indicating no signs, 1 indicating a few dilated vessels, 2 indicating numerous dilated vessels, and 3 indicating indistinct individual vessels.^18^ CECD was assessed with a specular microscope (EM‐3000, Tomey Corporation, Nagoya, Japan) (Figure 3D). All examinations and measurements were conducted by skilled ophthalmologists and orthoptists.

### Statistics

Data collected during the observational periods were analyzed using a mixed‐effects regression model, treating each participant's study ID as a random effect and the observation time as a fixed effect. Statistical significance was defined by a *p*‐value of <0.05. All statistical analyses were conducted with JMP Pro version 17.2.0 software (SAS Institute, Inc., Cary, NC, USA).

## RESULTS

Table 1 provides a summary of the collected data. Weekly room occupancy duration remained constant throughout the study (*p* = 0.38, mixed‐effects regression model). The baseline BCVA of −0.08 LogMAR (approximately 1.2 decimal visual acuity) in both eyes of participants showed minimal change, measuring −0.06 LogMAR (around 1.15 decimal visual acuity) in both eyes at the end of the study (*p* = 0.45 for both eyes). The mean SERE of −4.50 D in the right eye and −4.56 D in the left eye remained consistent at −4.59 D and −4.50 D, respectively, at the 36‐month follow‐up (*p* = 0.62 for the right eye and *p* = 0.71 for the left eye). Sample slit lamp photos and specular microscopy images for participant 2's left eye are shown in Figure 3. Throughout the observational period, all participants consistently recorded scores of 0 for corneal erosion area, density, and conjunctival hyperemia, with no observed cases of pterygium, cataract, or eyelid alterations. The baseline average CECD, initially 2689 cells/mm^2^ in the right eye and 2700 cells/mm^2^ in the left eye, remained stable at 2671 cells/mm^2^ and 2687 cells/mm^2^ at 36 months (*p* = 0.92 for the right eye and *p* = 0.99 for the left eye). No participants exhibited significant changes in any of the measured values. Additionally, none reported any subjective symptoms or experienced any systemic or ocular adverse effects.

**TABLE 1:** Observation results.

Parameter	Baseline	1 Day	1 Month	3 Months	6 Months	12 Months	36 Months	*p*‐value
Stay in the room (h/week)	7.3 ± 2.6	4.8 ± 0.5	7.3 ± 2.6	7.3 ± 2.6	7.3 ± 3.2	6.3 ± 3.5	5.5 ± 4.1	0.38
BCVA, LogMAR								
Right eye	−0.08	−0.08	−0.08	−0.08	−0.08	−0.08	−0.06 ± 0.04	0.45
Left eye	−0.08	−0.08	−0.08	−0.08	−0.08	−0.08	−0.06 ± 0.04	0.45
SERE, D								
Right eye	−4.50 ± 3.89	‐	−4.75 ± 4.14	−4.50 ± 3.81	−4.47 ± 3.79	−4.63 ± 3.81	−4.59 ± 4.05	0.62
Left eye	−4.56 ± 3.40	‐	−4.56 ± 3.43	−4.59 ± 3.47	−4.34 ± 3.29	−4.50 ± 3.42	−4.50 ± 3.70	0.71
Corneal erosion area score								
Right eye	0	0	0	0	0	0	0	‐
Left eye	0	0	0	0	0	0	0	‐
Corneal erosion density score								
Right eye	0	0	0	0	0	0	0	‐
Left eye	0	0	0	0	0	0	0	‐
Conjunctival hyperemia score								
Right eye	0	0	0	0	0	0	0	‐
Left eye	0	0	0	0	0	0	0	‐
Pterygium								
Right eye	None	None	None	None	None	None	None	‐
Left eye	None	None	None	None	None	None	None	‐
Cataract								
Right eye	None	None	None	None	None	None	None	‐
Left eye	None	None	None	None	None	None	None	‐
Lid change								
Right eye	None	None	None	None	None	None	None	‐
Left eye	None	None	None	None	None	None	None	‐
CECD, cells/mm^2^								
Right eye	2689 ± 187	‐	2654 ± 138	2717 ± 139	2630 ± 101	2672 ± 243	2671 ± 230	0.92
Left eye	2700 ± 145	‐	2668 ± 287	2724 ± 134	2713 ± 135	2725 ± 188	2687 ± 140	0.99
Subjective symptoms	None	None	None	None	None	None	None	‐
Adverse events	None	None	None	None	None	None	None	‐

*Note*: Data are expressed in mean ± standard deviation. *p*‐values are calculated by mixed‐effects regression model.

Abbreviations: BCVA, best‐corrected visual acuity; CECD, corneal endothelial cell density; D, diopter; LogMAR, logarithm of the minimum angle of resolution; SERE, spherical equivalent refractive error.

## DISCUSSION

In this clinical study, we extended our previous 12‐month observation period to evaluate the ocular safety of physicians working in an ophthalmic examination room equipped with 222 nm lamps, now tracking them for up to 36 months. The initial 12‐month study found no acute or long‐term effects on ocular health, with participants showing no evidence of photokeratitis or other UV‐related eye conditions.^16^ Findings at the 36‐month follow‐up remained consistent, as no clinically meaningful changes in ocular health indicators, including BCVA, SERE, and CECD, were detected among participants.

In previous studies, corneal epithelial damage and increased corneal light scattering were observed with UV‐C exposure at doses as low as 3.6 or 5.5 mJ/cm^2^, and more severe symptoms, including haze, granulation, and decreased visual acuity, were induced at doses of 10 mJ/cm^2^.^14^, ^19^ However, the exposure levels in our study, even under the assumption of the maximum possible exposure (13.2–16.5 mJ/cm^2^ if a person of 170 cm tall stood and directly gazed at the lamp for the whole duration), were still below Pitts' threshold for photokeratitis in humans (10 mJ/cm^2^) due to the real‐world conditions where physicians were seated most of the time and did not directly gaze at the lamps.^16^ In contrast to the earlier studies using 5000 W xenon‐mercury high‐pressure lamps, which had a wider monochromator band and introduced uncertainties due to stray light,^2^ our study used high‐output KrBr and KrCl excimer lamps with narrower emission spectra. Previous animal studies with these lamps demonstrated higher photokeratitis thresholds (15,000 mJ/cm^2^ for 207 nm and 5000 mJ/cm^2^ for 222 nm) in rats.^10^ Therefore, even at the maximum theoretical exposure levels in our study, the irradiation would not have been sufficient to cause photokeratitis.

In addition, our study sought to identify any potential delayed adverse effects of UV‐C exposure, such as pterygium, droplet keratopathy, cortical cataract, or eyelid skin malignancies—conditions that are typically associated with longer, deeper‐penetrating UV wavelengths.^20^, ^21^, ^22^, ^23^ At both the 12‐month and 36‐month follow‐ups, none of these conditions were observed, and key ocular health parameters (BCVA, SERE, CECD) remained stable. Extending the follow‐up period to 36 months further reinforces the conclusion that prolonged exposure to 222 nm UV‐C, within the parameters of this study, does not present a significant risk to ocular health in humans.^16^

The germicidal and pathogen‐inactivating effects of far UV‐C, including 222 nm wavelengths, have been extensively documented across various microorganisms. Prior studies have demonstrated its effectiveness against gram‐positive and gram‐negative bacteria, along with fungi and viruses in liquid suspensions.^24^, ^25^ It has also been shown to inactivate *E. coli* and P1 phages on cultured agar plates,^26^ SARS‐CoV‐2, which causes COVID‐19, in tissue cultures,^27^, ^28^ airborne H1N1 influenza virus,^29^ and human coronaviruses HCoV‐229E and HCoV‐OC43 in controlled experimental chambers,^30^ as well as aerosolized *S. aureus* in a large chamber setting.^6^ In animal models, 222 nm exposure effectively inhibited MRSA infections on mouse skin,^31^, ^32^ and in humans, it significantly reduced bacterial counts on skin exposed to suberythema doses in both healthy volunteers^33^ and patients with pressure ulcers.^34^ These findings emphasize the potential of 222 nm UV‐C for decontaminating surfaces in real‐world applications, suggesting further studies are needed.^27^ Recent studies indicate that a minimum dose of 27 mJ/cm^2^ of 222 nm UV‐C achieves over 95% germicidal efficacy against both gram‐negative and gram‐positive bacteria, whereas a dose of 25.1 mJ/cm^2^ achieves similar virucidal effectiveness against low‐pathogenic avian influenza virus and SARS‐CoV‐2.^35^ This current study focused on confirming the ocular safety of far‐UVC doses suitable for UVGI in humans. While it did not reevaluate the germicidal efficacy of the 222 nm lamps, our previous research confirmed their effectiveness, showing high inhibition rates for φX174 phage (>99%) and *S. aureus* (around 90%) on surfaces within the same full‐room UVGI setup as used in this study.^16^

In summary, our extended 36‐month evaluation of full‐room germicidal UV with 222 nm lamps revealed no health risks to the eyes or eyelid skin of individuals exposed to these conditions. These findings reinforce the safety of 222 nm UV‐C irradiation for ongoing use in occupied spaces, extending confidence beyond the initial 12‐month observation, and affirm its efficacy in microbial disinfection as shown in our prior study.^16^

## Data Availability

The data that support the findings of this study are available from the corresponding author upon reasonable request.
